# Forecasting the Effects of Fertility Control on Overabundant Ungulates: White-Tailed Deer in the National Capital Region

**DOI:** 10.1371/journal.pone.0143122

**Published:** 2015-12-09

**Authors:** Ann M. Raiho, Mevin B. Hooten, Scott Bates, N. Thompson Hobbs

**Affiliations:** 1 Natural Resource Ecology Laboratory, Department of Ecosystem Science and Sustainability, and Graduate Degree Program in Ecology, Colorado State University, Fort Collins, CO, 80523, United States of America; 2 U.S. Geological Survey, Colorado Cooperative Fish and Wildlife Research Unit; Department of Fish, Wildlife, and Conservation Biology and Department of Statistics, Colorado State University, Fort Collins, CO 80523, United States of America; 3 Urban Ecology Center, National Capital Region, National Park Service, Washington, D.C., United States of America; Centre for Cellular and Molecular Biology, INDIA

## Abstract

Overabundant populations of ungulates have caused environmental degradation and loss of biological diversity in ecosystems throughout the world. Culling or regulated harvest is often used to control overabundant species. These methods are difficult to implement in national parks, other types of conservation reserves, or in residential areas where public hunting may be forbidden by policy. As a result, fertility control has been recommended as a non-lethal alternative for regulating ungulate populations. We evaluate this alternative using white-tailed deer in national parks in the vicinity of Washington, D.C., USA as a model system. Managers seek to reduce densities of white-tailed deer from the current average (50 deer per km^2^) to decrease harm to native plant communities caused by deer. We present a Bayesian hierarchical model using 13 years of population estimates from 8 national parks in the National Capital Region Network. We offer a novel way to evaluate management actions relative to goals using short term forecasts. Our approach confirms past analyses that fertility control is incapable of rapidly reducing deer abundance. Fertility control can be combined with culling to maintain a population below carrying capacity with a high probability of success. This gives managers confronted with problematic overabundance a framework for implementing management actions with a realistic assessment of uncertainty.

## Introduction

Overabundant populations of wildlife can threaten human life or livelihoods, depress populations of other species, and harm ecosystem processes [1, 2]. Wildlife managers often seek to mitigate these effects by reducing the size of the problem population, traditionally by using regulated hunting or culling. Although hunting can provide recreational and economic benefits, lethal methods for regulating populations can be unpopular with the public or logistically infeasible because wildlife live in residential areas or in areas that prohibit hunting, for example in national parks and other types of conservation reserves [3]. Density dependence can make it difficult to control species by lethal means because reproductive rates can increase dramatically as a population declines [4]. Consequently, non-lethal methods, especially fertility control, have become more attractive to wildlife managers as a way to control wildlife populations [5, 6].

Analytical and simulation models cast doubt on the idea that fertility control can be used to efficiently achieve reduction goals for overabundant species or maintain populations within acceptable limits [7–10]. However, these models have been entirely deterministic and have rarely been combined with data. The ostensible certainty of the predictions of these models might create false confidence about decisions on fertility control. Thus, an important step in evaluating the efficacy of fertility control is to develop population models that are reliably assimilated with data to provide a statistically coherent assessment of uncertainty [11]. Models can provide a transparent and repeatable assessment of the feasibility of management alternatives by forecasting the effects of alternative actions on populations. Bayesian hierarchical models of population dynamics are a useful tool for evaluating management alternatives because they support forecasts accompanied by proper estimates of uncertainty.

White-tailed deer (*Odocoileus virginianus*) were once considered endangered but are now recognized as overabundant in many areas of North America [12]. In the late 19th century, there were fewer than 500,000 white-tailed deer in the entire United States. In 2000, there were over 30 million nationwide with high concentrations on the east coast of the United States [13]. Their feeding retards forest regeneration and harms biological diversity of vegetation by causing local extinction of many palatable understory plants [14, 15]. Overabundant deer threaten human safety by increasing traffic hazards and by providing a reservoir for ticks that carry lyme disease (*Lyme borreliosis*) and harm human economies by damaging crops [16].

Managers of National Parks in the eastern U.S. are especially concerned about the effects of overabundant wildlife on biological diversity because these effects are inconsistent with the mission of the parks. These concerns motivate an improved understanding of alternatives for population control. We developed a Bayesian state-space model to forecast the effect of alternative actions on white-tailed deer populations.

## Materials and Methods

The authority for data collection within National Parks rests with the National Park Service (NPS). All data for this study was provided by the National Capitol Region of the NPS. The National Park Service was a cooperator in this research. No animals were used in this research. All data was collected as a matter of routine park management duties and analyzed retrospectively for this study.

### Population and Classification Data

**Fig 1:**
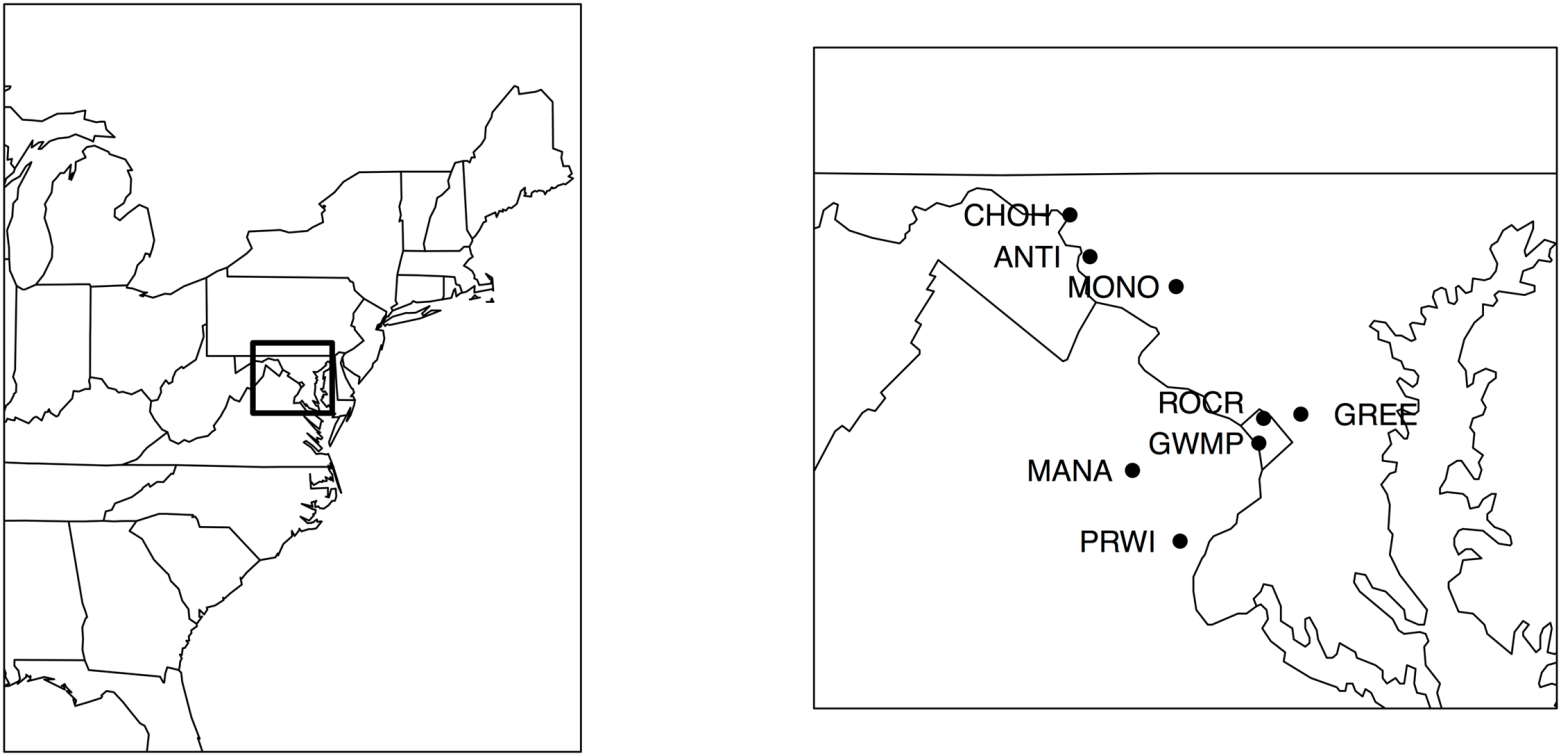
The study area included eight parks in the National Capital Region Network in the area surrounding Washington D.C. Each park used standardized distance sampling methods which produced a time series from 2000 to 2011 of regional estimates of white-tailed deer abundance.

We analyzed observations of white-tailed deer population density and composition from eight National Parks in the National Capitol Region Network (NCRN) near Washington, D.C. during 2001–2013. The NCRN parks have collaborated using standardized distance sampling methods [17] to annually obtain density estimates of white-tailed deer populations in each park (Fig 1). The authority for data collection within National Parks rests with the National Park Service (NPS). All data for this study was provided by the National Capitol Region of the NPS.

*Distance Sampling Protocol*.— Distance sampling is a widely used approach for estimating the abundance of wildlife populations [17]. A standard operating procedure for distance sampling of deer was used for all eight parks in the NCRN [18]. Surveys were conducted after leaf-fall and before hunting season for three consecutive nights in each park. Three observers identified deer on specified transect roads with a spotlight to locate groups of deer. After the deer were counted, the distance from observers was determined with a laser rangefinder positioned perpendicular to the transect. Observers also classified each deer in every group as buck, doe, fawn, or “unknown.” Each night was treated as a replicate, and the data were pooled for analysis.

*Density Estimate Analysis*.— Program Distance [19] was used to convert these distance sampling counts to estimates of mean population densities with an associated standard error for each park each year. The detections were divided into 10–12 even distance intervals. Intervals were expanded, narrowed, or dropped from the analysis to produce a smooth shoulder as the distance from the observer to the deer increased. A smooth shoulder prevents heaping of data into one distance interval, and corrects for outliers caused by evasive movements [20]. Once a satisfactory shoulder was produced, four models were fit to the data (uniform, half-normal, hazard rate, and negative exponential). The three criteria used to choose the best fitted model were: (1) percent coefficient of variation (CV) less than 20; (2) the detection probability variation was less than 30%; and (3) lowest Akaike’s Information Criterion (AIC) score. Program Distance calculates all three measures. These procedures resulted in a density estimate with related standard error for each park each year. Explicit protocol for using Program Distance to calculate density is included in the Supplemental Materials. We used density estimates from 8 parks over 13 years in our analyses.

### Analysis

We used a fully Bayesian, hierarchical model to obtain posterior distributions of parameters, latent states, and derived quantities of interest. Berliner [21] provides a unified framework for Bayesian, hierarchical models of time series. Our hierarchical model contains: a model of the ecological process, two models linking the process to data, and models for parameters. In the sections that follow, we describe these models.

### Process Model

We developed a Lefkovitch matrix [22] to predict the median number of individuals in three stages at population survey; juveniles of both sexes aged 0 to 6 months (*n*_1_), adult females, aged ≥ 1.5 years and older (*n*_2_), and adult males aged ≥ 1.5 years and older (*n*_3_). Model census [23] occurs six months after the birth pulse. The deterministic model is

$$\begin{array}{ccc} {\text{A}}_{it} & = & \left[\begin{array}{ccc} 0 & s_{2i}f_{it} & 0\\ s_{1i}m & s_{2i} & 0\\ s_{1i}\left(1-m\right) & 0 & s_{3i} \end{array}\right] \end{array}$$ (1)

$$\begin{array}{ccc} {\text{n}}_{it} & = & {\text{A}}_{it}{\text{n}}_{it-1}, \end{array}$$ (2)

where *i* indexes parks and *t* indexes years. Thus, **n**_**it**_ is the vector of the true, unobserved mean number of animals in each age/sex class in park *i* at time *t*. Parameters in the projection matrix **A** are survival probabilities for juveniles (*s*_1*i*_), females (*s*_2*i*_), and males (*s*_3*i*_), fecundities *f*_*it*_ for each park (*i*) and year (*t*), and sex ratio (*m*). These survival probabilities include effects of harvest because we lacked data on harvest mortality at the scale of our analysis. Survival probabilities were not raised to a power [24] because the preponderance of mortality occurs after census and before birth pulse due to the combined effects of winter severity and harvest. Fecundities implicitly include survival of juveniles from the birth pulse to census. We accounted for effects of population density on fecundity [25] using

$$\begin{array}{c} f_{it}=e^{r_{f}-\frac{r_{f}}{K_{f}}(\sum_{j=1}^{3}n_{jit-1}/{\text{area}}_{i})}, \end{array}$$ (3)

where *r*_*f*_ is the maximum number of fawns surviving to census produced per doe when population size is zero and *K*_*f*_ is the density of of animals when fecundity equals zero.

We included stochasticity in the process model to account for influences on the true state that are not represented in the deterministic model:

$$\begin{array}{ccc} \text{log}({\text{n}}_{it}) & ∼ & \text{multivariate}\;\text{normal}(\log({\text{A}}_{it-1}{\text{n}}_{it-1}),\sigma_{p}^{2}\text{I}), \end{array}$$ (4)

where $\sigma_{p}^{2}$ is the process variance, a parameter that represents all sources of variation in deer abundance that are not included in our deterministic model. We assumed a single process variance for all stages and parks.

### Parameter Models

We assumed that parameters representing survival probabilities for each park were drawn from a common distribution. This approach allowed us to estimate survival probabilities for each park (*s*_*ij*_) while summarizing the survival probabilities across the region (*μ*_*j*_) defined by hyper-parameters of the distribution from which the park-specific survival probabilities were drawn (*a*_*j*_ and *b*_*j*_).

$$\begin{array}{ccc} s_{ij} & ∼ & \text{beta}(a_{j},b_{j})\\ a_{j} & ∼ & \text{gamma}(\frac{\mu_{a_{j}}^{2}}{\sigma_{a_{j}}^{2}},\frac{\mu_{a_{j}}}{\sigma_{a_{j}}^{2}})\\ b_{j} & ∼ & \text{gamma}(\frac{\mu_{b_{j}}^{2}}{\sigma_{b_{j}}^{2}},\frac{\mu_{b_{j}}}{\sigma_{b_{j}}^{2}})\\ \mu_{j} & = & \frac{a_{j}}{a_{j}+b_{j}} \end{array}$$

where *j* indexes age-sex class. Hyper-parameter distributions were informed from a group level meta-analysis of survival probabilities reported in the literature.

We assumed that juvenile deer survived to the adult stage with a 1:1 sex ratio (*m*) with a variance of 0.02 such that by moment matching

$$\begin{array}{c} m∼\text{beta}(312,312). \end{array}$$

This assumption was made because there will not be preferential harvest during a juvenile’s first year. We chose a variance of 0.02 because it is less than 5% of 0.5 and allowed a small amount of variation.

We used an allometric equation for the scaling of birth rate of Artiodactyls [26] to inform the prior distribution of *r*_*f*_ (Eq 3),

$$\begin{array}{c} r_{f}∼\text{normal}(2\cdot 3.09w^{-0.33},0.1304^{2}). \end{array}$$

We assumed a mean body mass (w) of 65 kg for white-tailed deer [25] and calculated a residual standard error of 0.1304. We multiplied Western’s equation by 2 because his model is offspring per individual in the population including males and females. To ensure that our use of Western’s equation was reasonable, we used it in a simple, deterministic 2 × 2 matrix model with adult survival set at 0.90. We then compared the prediction of population growth rate (*λ*_Western_) based on this matrix model with the prediction of *λ* obtained from the scaling relationship of [27] (*λ*_Sinclair_). There was close agreement between the two predictions (*λ*_Western_ = 3.09*w* ^−0.33^, *λ*_Sinclair_ = 1.375*w* ^−0.315^).

Properly specified, dynamic, statistical models require estimating initial conditions as parameters. The initial conditions of the state vector **n**_*i*1_ were informed by both types of data using

$$\begin{array}{ccc} \gamma_{i1} & ∼ & \text{Dirichlet}({\text{y}}_{\alpha_{i1}}+1) \end{array}$$ (5)

$$\begin{array}{ccc} d_{i1} & ∼ & \text{normal}(y_{d_{i1}},{\hat{\sigma }}_{i1}^{2}) \end{array}$$ (6)

$$\begin{array}{ccc} n_{i1} & = & d_{i1}\times \gamma_{i1}\times {\text{area}}_{i}, \end{array}$$ (7)

where ***y***_***α***_*i*1__ are the categorical data for each park at year 1, *y*_*d*_*i*1__ are the density data for each park at year 1 with associated standard error (${\hat{\sigma }}_{i1}$) from Program Distance [19], and ***n***_*i*1_ represents the initial conditions for each park at year 1.

### Data Models

Likelihoods were composed using two sources of data described in detail above, observations of sex and age structure (**y**_***α***_*it*__), as well as density data (*y*_*d*_*it*__), for each park (*i*) at each year (*t*). The vector **y**_***α***_*it*__ represents the number of animals classified as juvenile, adult female and adult male for park *i* at time *t*, and *y*_*N*_*it*__ is the total number of animals categorized. The likelihood for the classification observations was

$$\begin{array}{c} y_{\alpha_{it}}∼\text{multinomial}\left(y_{N_{it}},\pi_{it}\right), \end{array}$$

where ***π***_*it*_ is a vector of proportions from the process model

$$\begin{array}{c} \pi_{it}=[\frac{n_{1it}}{\sum_{j=1}^{3}n_{jit}},\frac{n_{2it}}{\sum_{j=1}^{3}n_{jit}},\frac{n_{3it}}{\sum_{j=1}^{3}n_{jit}}]\;. \end{array}$$ (8)

We estimated the population density for each park by dividing the estimated total deer abundance for each park by the parks area (Eq 9). Animal density *y*_*d*_*it*__ and a standard error ${\hat{\sigma }}_{it}$ for each park and year were estimated in prior analyses by park managers using Program Distance as described in section *Density Estimate Analysis* [18, 19]. We used these data in the likelihood

$$\begin{array}{ccc} y_{d_{it}} & ∼ & \text{normal}(\frac{\sum_{j=1}^{3}n_{jit}}{{\text{area}}_{i}},{\hat{\sigma }}_{it}^{2}), \end{array}$$ (9)

where *area*_*i*_ is the total area of park *i*. We show the posterior and joint distributions for the full model in Appendix A in S1 File.

*Estimation*.—Marginal posterior distributions of states, parameters, and model predictions were calculated using Markov chain Monte Carlo (MCMC) methods implemented in JAGS 3.3.0 [28, 29] in the R 3.0.2 computing environment [30] using the ‘rjags’ package [31]. Initial values of chains were chosen to be diffuse to the means of the prior distributions [32]. We accumulated 40,000 samples from each chain following a 8,000 iterations as burn-in. Convergence was assessed by inspection of trace plots and by standard diagnostics of [32] and [33].

*Model Evaluation*.—We tested for lack of fit using posterior predictive checks. This approach compares data simulated from the model to real data used to estimate the model parameters [34]. If the distribution of the simulated data fails to resemble the distribution of the real data, there may be structural deficiencies in the process or the data models. We calculated a test statistic from the observed data (*T* ^*obs*^) and from the simulated data sets (*T* ^*rep*^),

$$\begin{array}{ccc} T^{obs}=\sum_{i=1}^{I}\sum_{t=1}^{T}{\left(y_{d_{it}}-\mu_{it}\right)}^{2} & & T^{rep}=\sum_{i=1}^{I}\sum_{t=1}^{T}{\left(y_{d_{it}}^{rep}-\mu_{it}\right)}^{2}, \end{array}$$

where $y_{d}^{rep}$ is drawn from the posterior predictive distribution and *μ*_*it*_ is the model prediction of the median of the distribution of the density of white-tailed deer in each park, each year.

We then calculated a Bayesian P-value,

$$\begin{array}{ccc} P_{B} & = & Pr[T^{rep}\left(y^{rep},\theta \right)\geq T^{obs}\left(y,\theta \right)|y]. \end{array}$$

Lack of fit is indicated if *P*_*B*_ is close to 0 or 1.

### Model Experiments

We conducted model experiments by adding an additional sterile class of adult female deer to our matrix representation of population dynamics. We implemented four treatments: culling, sterilization, one-year fixed duration contraceptives, and three year average duration contraceptives. These treatments were administered at four levels: 20%, 40%, 60%, or 90% of adult females were treated each year to allow direct comparison among treatment type and treatment intensity. Fawns were not given contraceptives in these experiments because fawns do not have a large effect on population birth rate [35]. We assumed that culling, sterilization, or contraceptives were administered immediately after census. These experiments were chosen to represent a variety of intensity from worst to best case management scenario. Details on the simulation study, including changes made in the process model for each experiment, can be found in Appendix B in S1 File.

*Eigenanalysis*.—The equivariance property of MCMC means that quantities calculated from random variables are random variables with their own posterior distributions [36]. We sought inference on the effects of treatment on the long-term population growth rate calculated from linear projection matrices containing random variables. The dominant eigenvalue describes the ergodic properties of population growth [23]. We linearized projection matrices by setting *N*_*it*−1_ = 0 in Eq (3). We calculated the posterior distributions of asymptotic growth rate (*λ*) by performing a classic eigenanalysis using the MCMC output. A single sample of *λ* was calculated from the projection matrix at each iteration in the chain. Growth rates calculated this way provide an upper bound on the effect of each treatment using the estimated maximum possible fecundity in the analysis. The population growth rate calculated this way is useful for comparison between model experiments because it will use the same scale to show the effect of management on population growth. The eigenanalysis was implemented with package ‘popbio’ [37] in the R computing environment [30].

**Fig 2:**
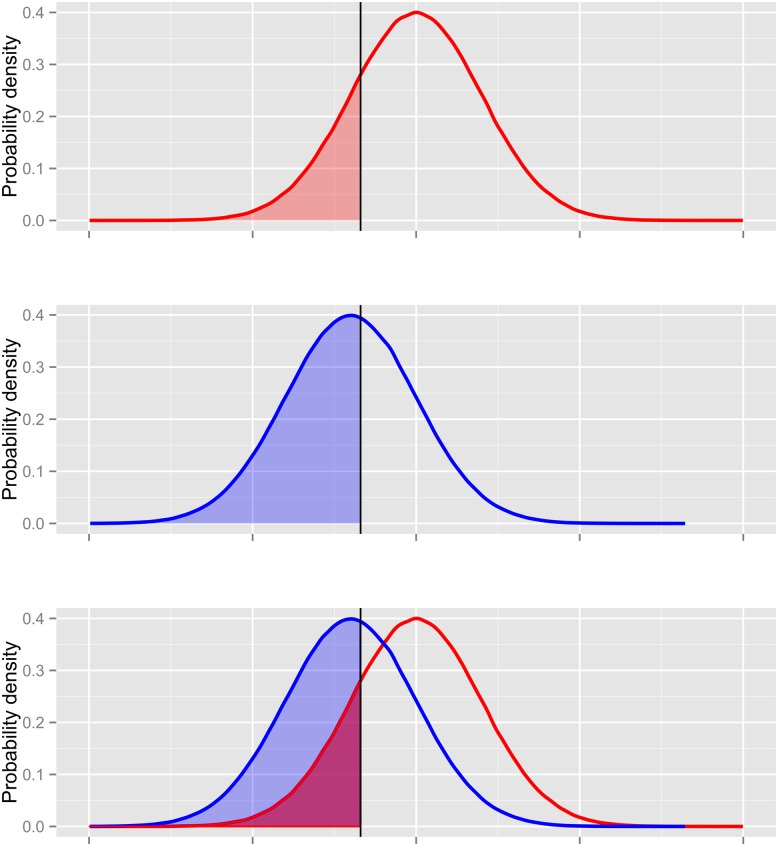
A. The vertical line indicates a manager’s objective for the population. The area that is shaded red indicates the probability that an objective will be met given no action. B. The Posterior distribution conditional on a management action, for example, culling or delivering contraceptives. The blue shaded area under the curve is the probability that a manager will reach the objective given this action. C. The net effect of management is the ratio of the blue shaded area to the red shaded area.

*Evaluating Management Action*.—We calculated posterior predictive process distributions for future states using

$$\begin{array}{ccc} \left[n_{T+1}|y_{1},...,y_{T}\right] & = & \int \int ...\int [n_{T+1}|n_{T},\theta_{m},\sigma_{p}^{2}]\\ & & [n_{1}...,n_{T},\theta_{1},...,\theta_{m},\sigma_{p}^{2}|y_{1},...,y_{T}]d\theta_{1},...,d\theta_{m},d\sigma_{p}^{2}dn_{1},...,dn_{T}, \end{array}$$

where ***n***_*T*+1_ is the true state of the population in the future given the data (*y*_1_, …, *y*_*T*_). We assessed the effectiveness of different treatments [38] by calculating the probability that a specific goal would be met given the treatment relative to no action. We first obtained the posterior predictive process distribution of the true state of the population at a point in the future and used Monte Carlo integration to approximate the probability that the goal will be met *given no action*. (Fig 2A). We repeated this procedure assuming a treatment was applied to the population. The ratio of the probability that an objective will be realized given implementation of a treatment over the probability that an objective will be realized given no action was used to assess the net effect of treatment in the face of uncertainty. (Fig 2C). This net effect can be reported to a manager in terms such as, “You are ten times more likely to achieve your goal if you implement this treatment relative to taking no action.”

## Results

### Model Checking and Parameter Estimation

We verified the MCMC algorithm by simulating data and comparing the means of recovered parameters with the parameter values used to simulate the data indicating convergence of the MCMC [39, 40]. The upper quantile of Gelman-Rubin diagnostics [41] was ≤ 1.01 for all parameters. Posterior predictive checks showed no evidence of lack of fit (See Appendix C: Fig B in S1 File).

**Fig 3:**
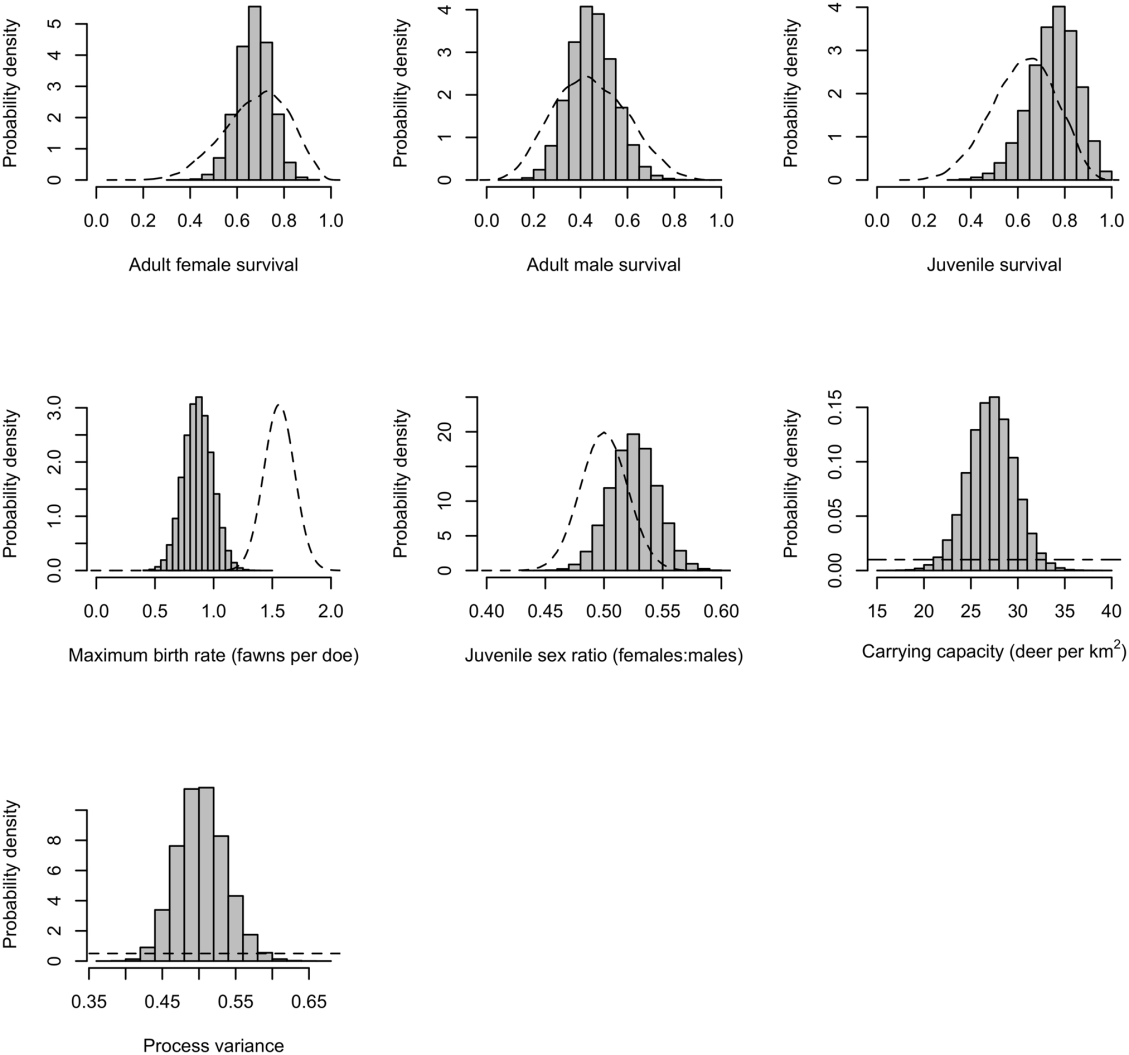
Posterior (bars) and prior (dashed lines) distributions for vital rate parameters. One set of parameters was estimated for all parks.

**Fig 4:**
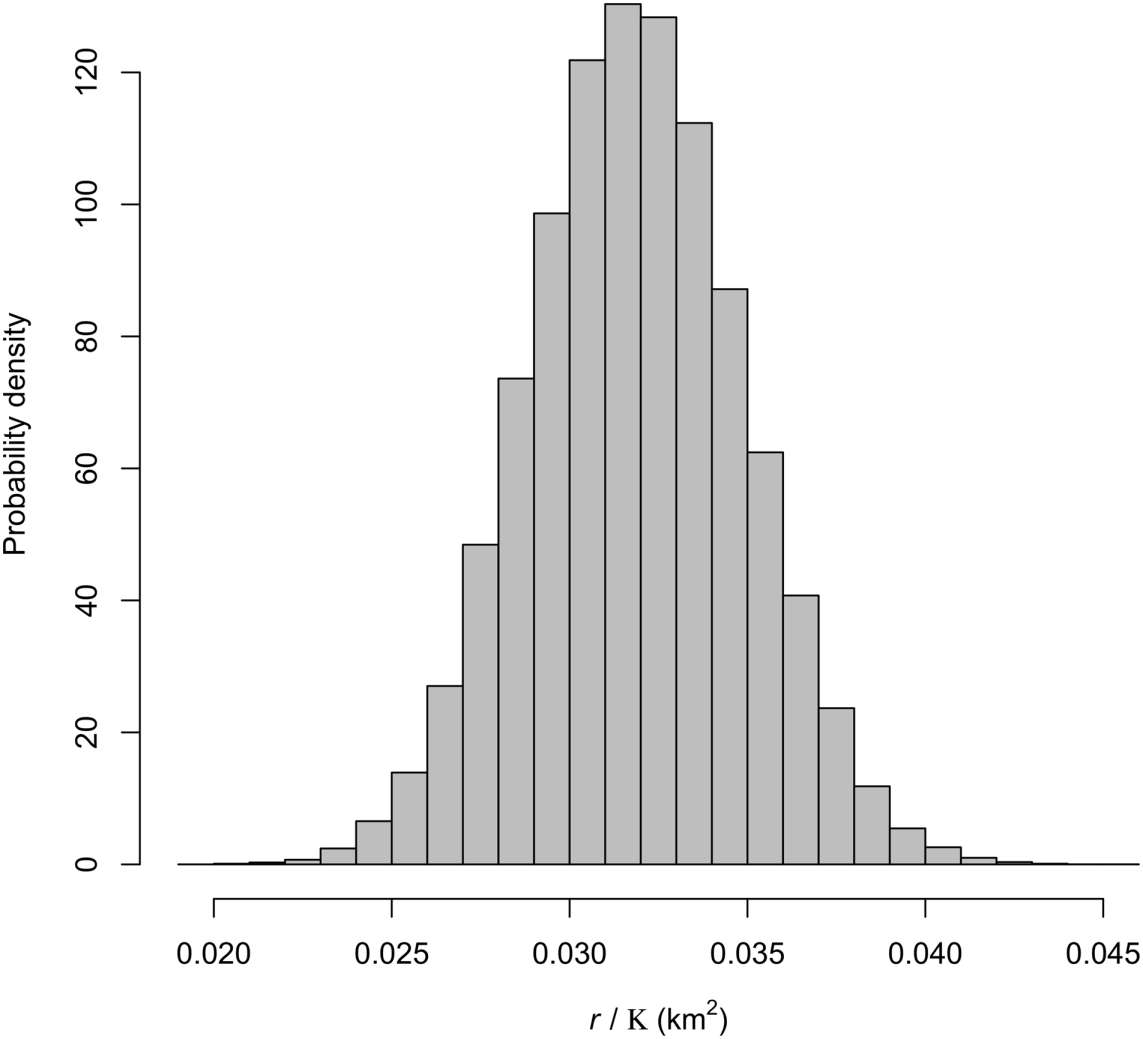
The posterior distribution of the slope (*r*/*K*) of the density dependent function.

Survival probability posterior distribution of adult females broadly overlapped the survival probability posterior distribution of juveniles (Table 1, Fig 3). We estimated that 0.86 fawns surviving to census would be produced per adult female at low population density. This result is much less than our prior (mean = 1.56, SD = 0.1304). We observed a negative feedback between population density and number of fawns produced per female (Fig 4). The matrix model was most sensitive to adult female survival. The posterior distributions of the sensitivities of the elements of the matrix can be found in Appendix C in S1 File.

**Table 1:** Estimates of model parameters and 95% equal tailed Bayesian credible intervals (BCI).

	Mean	Median	SD	2.5% BCI	97.5% BCI
Carrying capacity (*K*_*f*_ deer per km^2^)	27.12	27.13	2.52	22.11	32.06
Maximum fecundity (*r*_*f*_)	0.86	0.86	0.12	0.62	1.11
Juvenile sex ratio (m)	0.53	0.53	0.02	0.49	0.56
Female survival probability (*s*_2_)	0.67	0.68	0.07	0.53	0.81
Male survival probability (*s*_3_)	0.45	0.45	0.10	0.27	0.65
Juvenile survival probability (*s*_1_)	0.75	0.76	0.10	0.54	0.93
Process variance (*σ* ^2^)	0.50	0.50	0.03	0.44	0.57

### Forecast without Management Action

**Fig 5:**
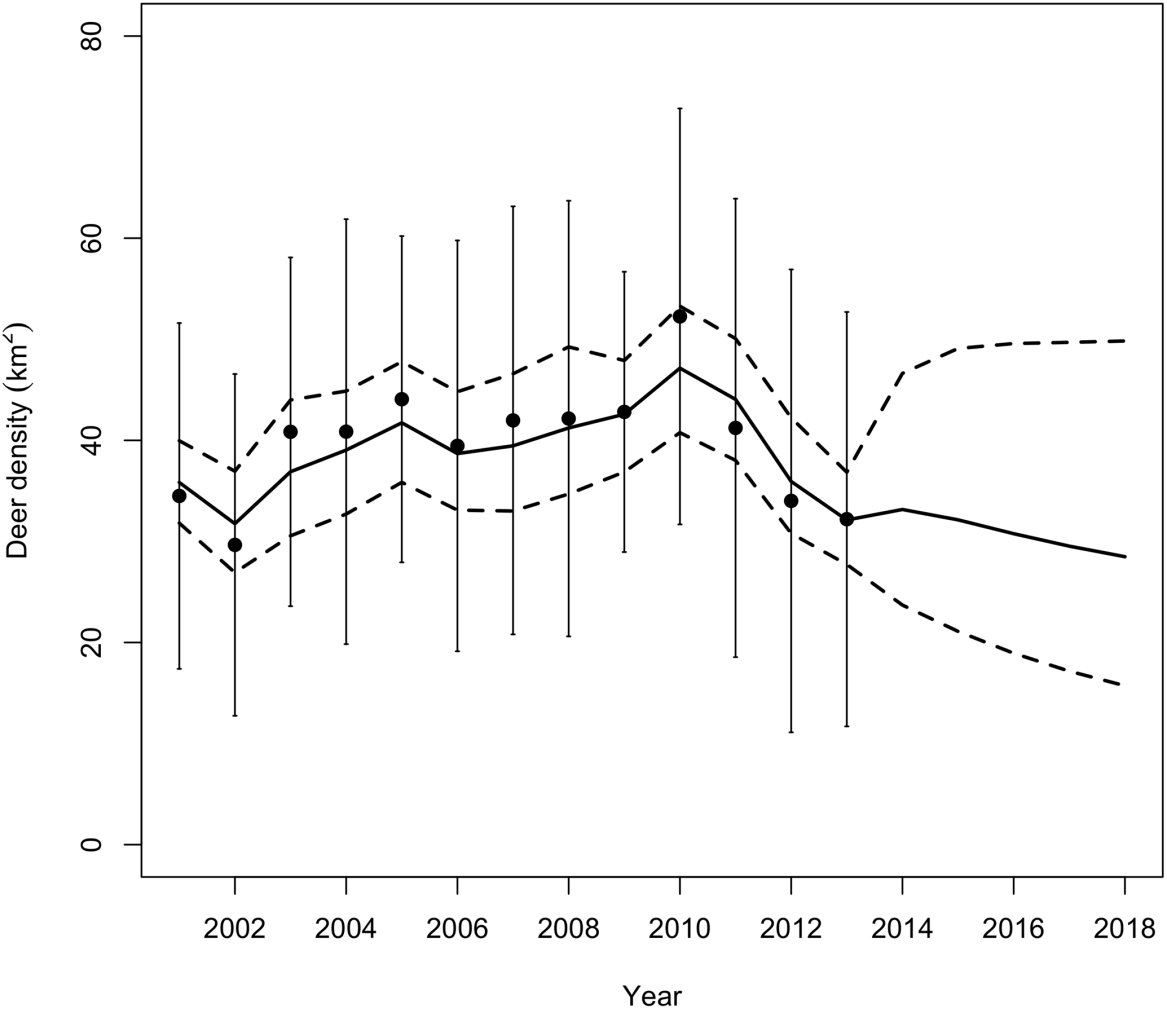
Estimate of the population density of white-tailed deer in the National Capital region during 2001 to 2013 and forecasts from 2014 to 2018. The solid line represents the median of the posterior distribution. Dotted lines show the 95% equal-tailed Bayesian credible intervals. The vertical lines are ± one standard deviation of the means of the density data.

**Fig 6:**
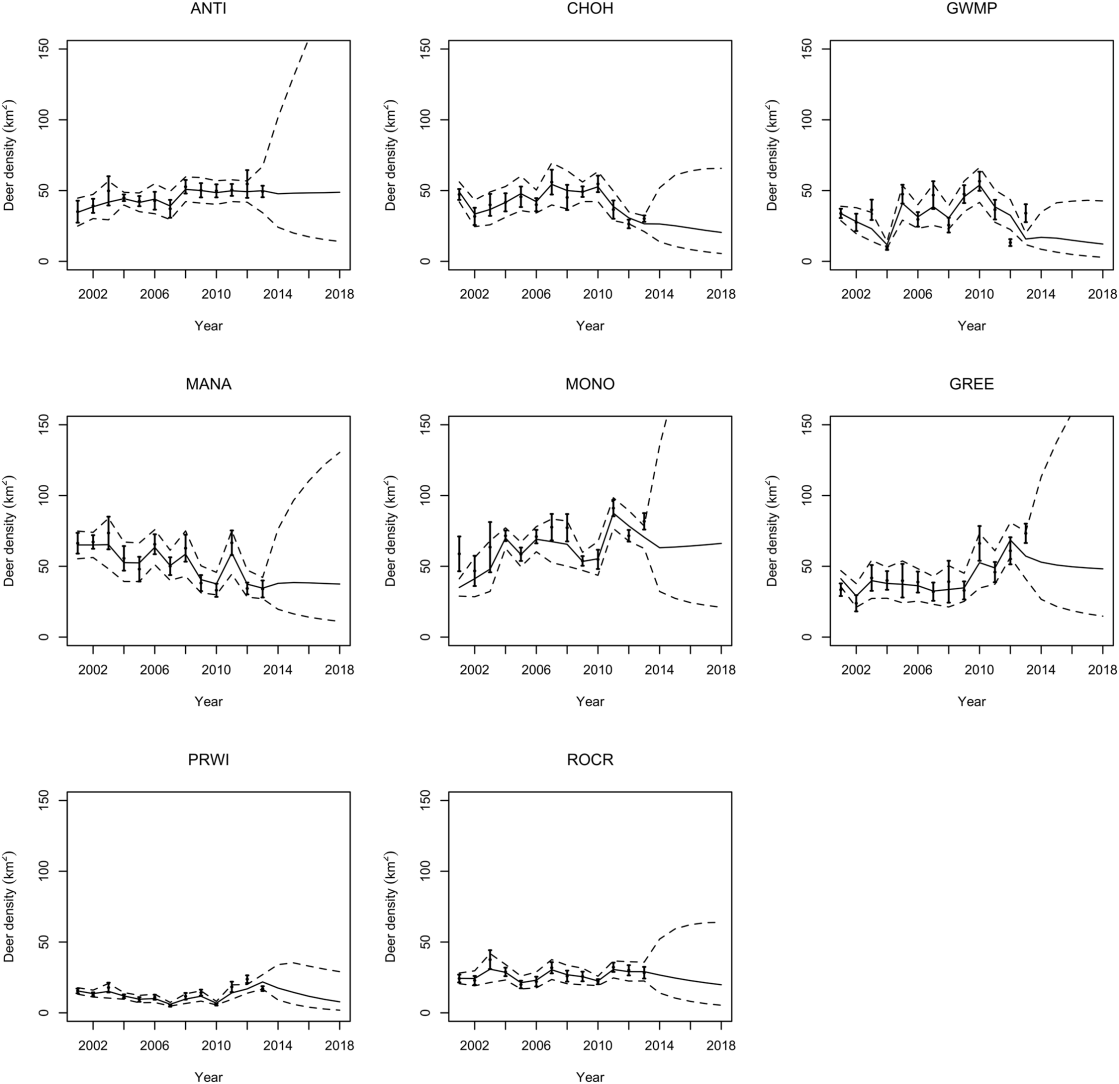
Forecast by park for the years 2001 to 2018. The solid line is the median of the estimate. The dashed lines are the 95% Bayesian credible intervals (BCI) of the estimate. The dots with vertical lines are the medians of the data with standard deviation error bars. The abbreviations for each park are in the titles of each plot. ANTI: Antietam National Battlefield, CHOH: Chesapeake and Ohio Canal NHP, GWMP: George Washington Memorial Parkway, MANA: Manassas National Battlefield Park, MONO: Monocacy National Battlefield, GREE: Greenbelt Park as part of the National Capital Parks East, PRWI: Prince William Forest Park, ROCR: Rock Creek Park.

**Fig 7:**
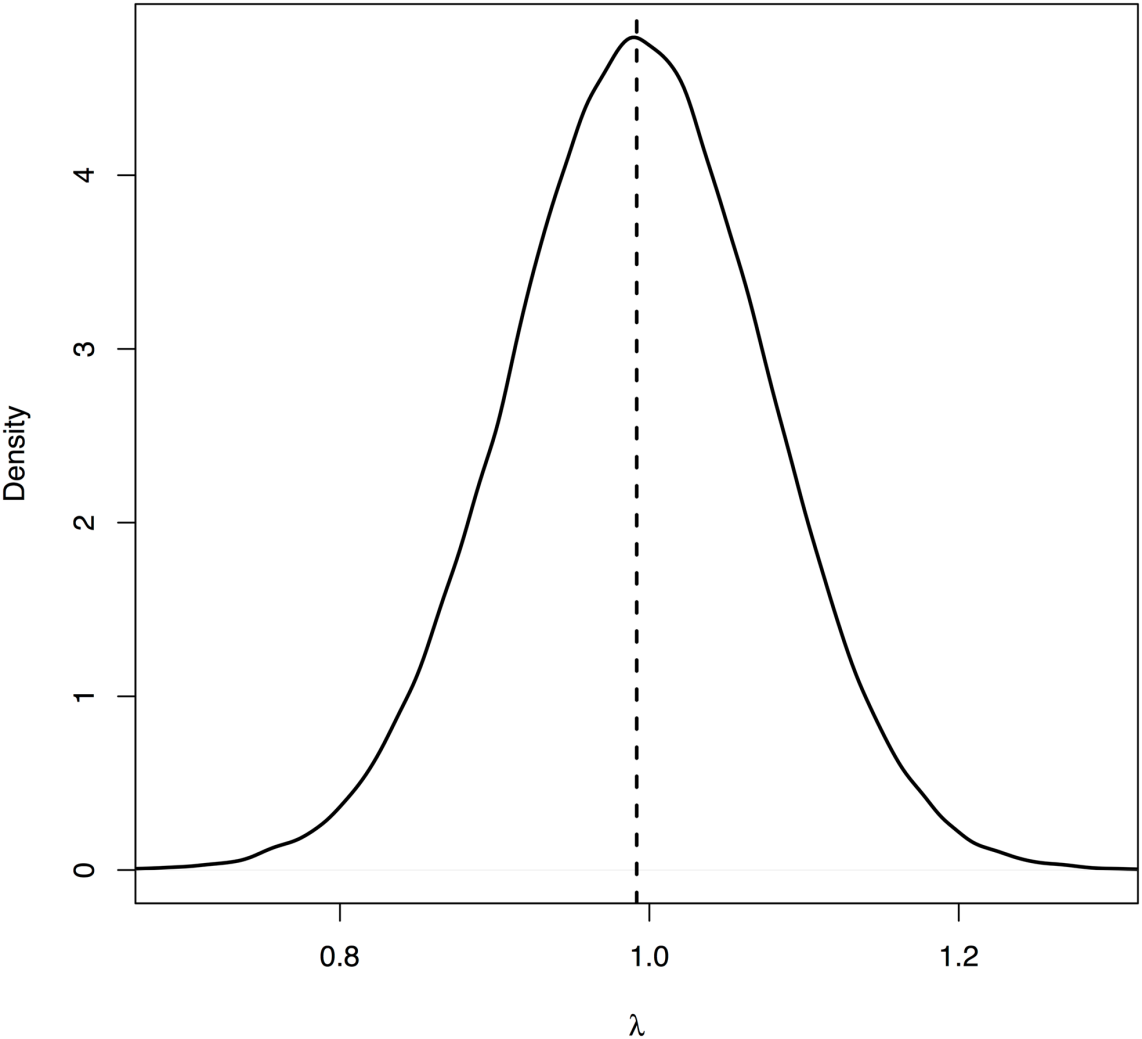
Population growth rate (*λ*) of the white-tailed deer in the National Capital Region without any management action has a median of 0.992 (95% equal tailed Bayesian credible interval, BCI = 0.824,1.16). The median suggests that the population is currently declining. However, the posterior distribution overlaps 1, suggesting we cannot rule out the possibility that it is increasing.

We made regional forecasts for white-tailed deer density from 2014 to 2018 (Fig 5) as well as forecasts for each park (Fig 6). The posterior distributions of population size indicated the population declined during 2001–2013 with a median population growth rate of 0.992 (95% equal tailed Bayesian credible interval, BCI = {0.824, 1.16}) (Fig 7). The median of the posterior process distribution suggests these declines will continue through 2018. However, the wide credible intervals on forecasts indicate substantial uncertainty about the growth of the population in the future. We cannot rule out the possibility that the population will increase in the future (Fig 5).

### Results of Model Experiments

We report the effects of different fertility control regimes on *λ* and the probability of achieving a hypothetical management objective. We also show the estimated number of adult female deer that would need to be treated over five years in the region in order to achieve the reported effect on *λ*, which we will refer to as “treatment numbers.” Model experiments can be compared on three different levels: type of treatment, percent treated, and treatment number.

**Fig 8:**
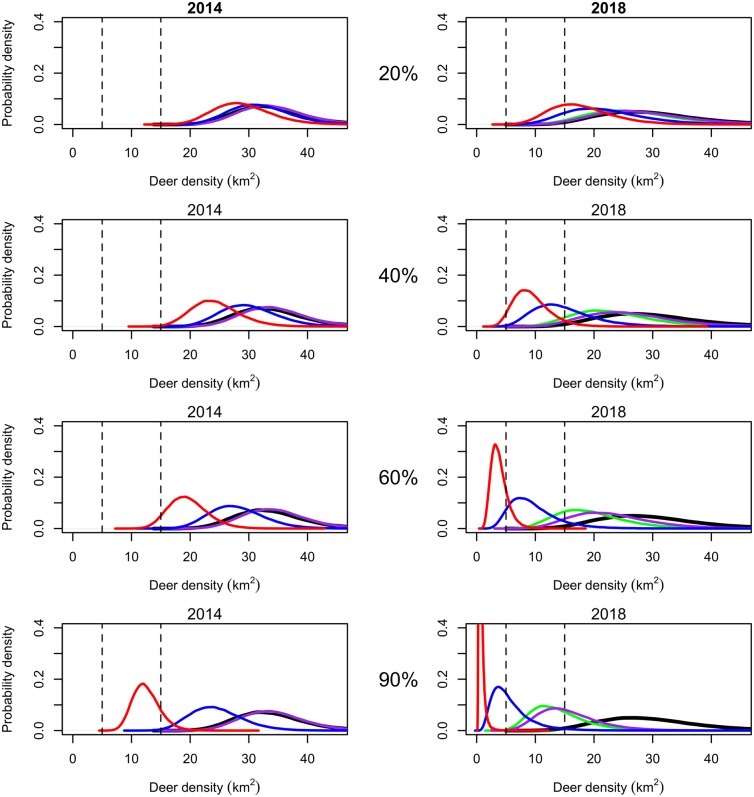
Effects of fertility control treatments on white-tailed deer population densities forecasted for 2014 and 2018 with plot rows corresponding to percent treated from top to bottom: 20%, 40%, 60%, and 90%. The black distribution represents the change in population with no action to show that the population may decrease without any management action. The dashed vertical lines represent the bounds of the hypothetical management objective. The purple distribution represents the population effect of contraceptives with one year effectiveness. The green distribution represents the population effect of contraceptives with three year effectiveness. The blue distribution represents sterilization. Sterilization reduced the population at a faster rate than contraceptives. Culling (the red distribution) had the most dramatic effect on population density.

**Fig 9:**
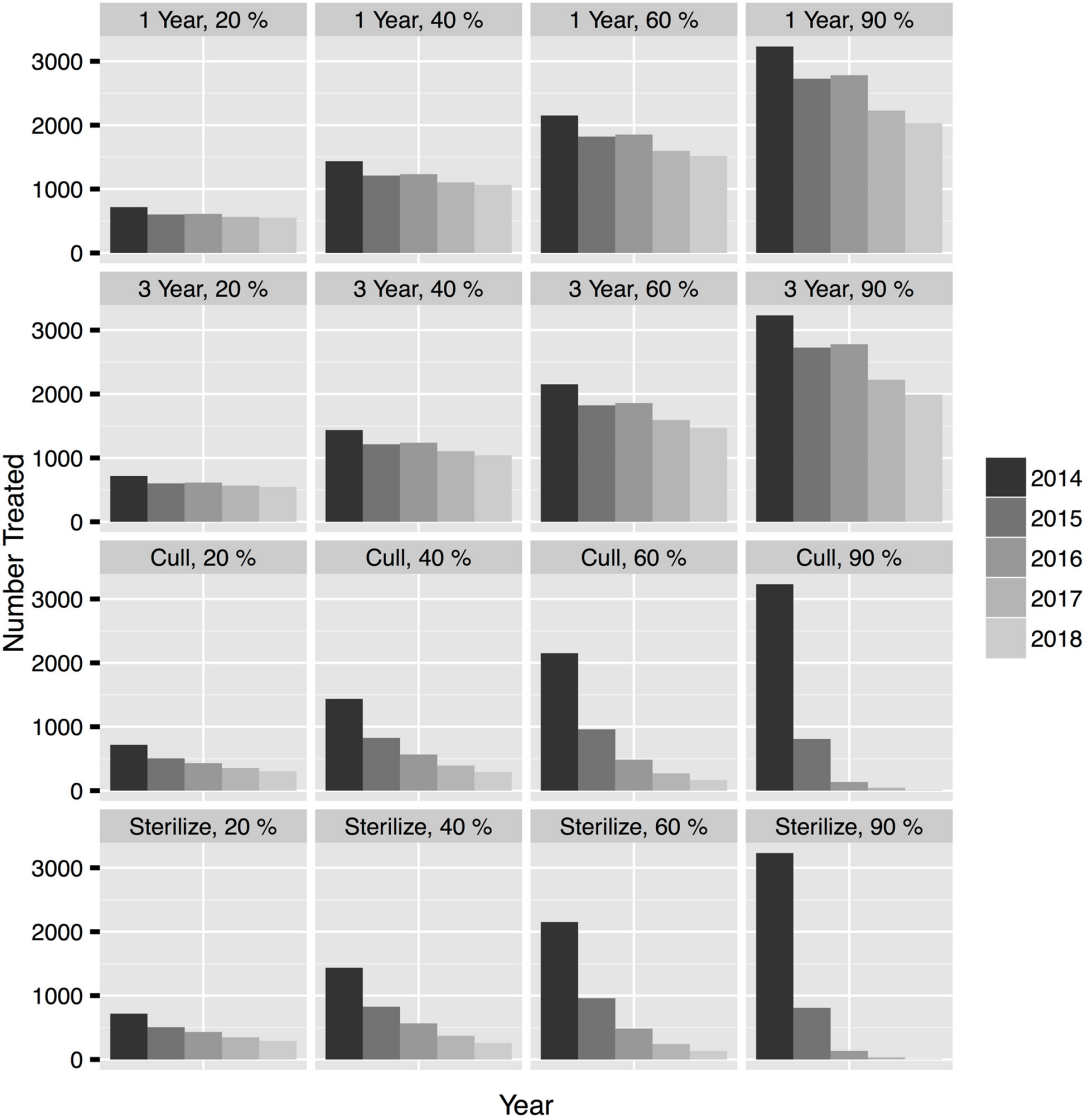
The median number of adult females that would need to be treated across all parks for each management scenario over five years.

*Growth rate* *and treatment numbers*.—We calculated the growth rate for each treatment to compare treatment effects. All treatment types and intensities reduced *λ* compared to no action (Table 2). Our most extreme culling experiment, culling 90% of adult females, reduced the population growth rate from 0.99 to 0.21 (median difference = 0.78, 2.5% BCI on difference = 0.63, 95% BCI on difference = 0.95) and required moderately high treatment numbers (≈ 4,200 over 5 years). Culling and sterilization required similar treatment numbers at all levels of implementation. Sterilization of any proportion of adult females reduced the median population growth rate to about 0.7 assuming sterilization was 100% effective. Values of *λ* are similar at every treatment level because *λ* reaches the ergodic growth rate of the population when all females are sterile. Contraceptives had the smallest effect on growth rate and much higher treatment numbers especially when treating higher percentages of adult female deer (Figs 8 and 9). Treating 90% of the adult female population with contraceptives lasting one year assuming 100% effectiveness or three years assuming a three year average effectiveness had almost the same effect on *λ* as sterilization, but had a much higher treatment number (≈ 13,000 over 5 years). The short duration contraceptives had the smallest effect of any treatment relative to no action when 20% were treated each year (median difference = 0.034, 2.5% BCI on difference = −0.16, 97.5% BCI on difference = 0.26), and the treatment numbers were comparable to culling 40% (≈ 3,500 over 5 years).

**Table 2:** Population growth rates and the median difference of an action relative to no action (RNA) for different fertility control regimes.

Treatment	Percent Treated	2.5% BCI	Median	97.5% BCI	2.5% BCI RNA	Median Difference RNA	97.5% BCI RNA
No Action	0	0.82	0.99	1.16			
Cull	20	0.70	0.84	0.98	−0.03	0.15	0.37
Sterilize		0.70	0.84	0.98	−0.03	0.15	0.37
1 Year		0.79	0.96	1.12	−0.16	0.03	0.26
3 Year		0.77	0.93	1.08	−0.13	0.07	0.29
Cull	40	0.57	0.68	0.80	0.14	0.31	0.51
Sterilize		0.58	0.70	0.82	0.12	0.30	0.50
1 Year		0.76	0.92	1.07	−0.11	0.07	0.30
3 Year		0.72	0.86	1.01	−0.05	0.13	0.35
Cull	60	0.43	0.51	0.60	0.32	0.48	0.66
Sterilize		0.53	0.68	0.81	0.14	0.32	0.54
1 Year		0.72	0.87	1.01	−0.06	0.12	0.34
3 Year		0.67	0.80	0.94	0.01	0.19	0.40
Cull	90	0.18	0.21	0.25	0.63	0.78	0.95
Sterilize		0.53	0.67	0.81	0.14	0.32	0.54
1 Year		0.63	0.76	0.89	0.05	0.23	0.44
3 Year		0.58	0.71	0.84	0.10	0.28	0.49

*Probability of achieving a management objective*.—We evaluated the feasibility of maintaining the white tailed deer population density between 5 and 15 deer per km^2^ using different management actions by calculating the probability that a treatment would cause the population to be below, within, or above the objective 5 years into the future with methodology explained in section *Evaluating Management Action* (Fig 2 and Table A in S1 File). There was a small chance (2%) that the population would be within the objective in five years without management action because the median population density was forecasted to decline in the absence of any treatment. The only management regimes that had a probability > 0.01 of achieving the objective the first year was culling 60% or 90% of the adult female population (Fig 8). However, continuing to cull at these levels each year had a high probability of pushing the population below the lower bound of the objective potentially driving the population locally extinct (Table A in S1 File). Culling 40% of the population for 5 years had a high probability of maintaing the population within the objective range (Probability in = 0.91) while culling only 20% for 5 years had a lower probability of being within the objective (Probability in = 0.32). As suggested by the derivation of population growth rates above, culling reduced the population most quickly followed by sterilization. Sterilization had the highest probability of meeting the management objectives when 60% of the adult females were treated for 5 years (Probability in = 0.81). Contraceptives had the smallest probabilities of meeting the objective (Probability in ≤ 0.65 for all proportions for all years). However, treating 90% with 3 year contraceptives for five years was 40 times more likely to achieve the objective than no action.

**Fig 10:**
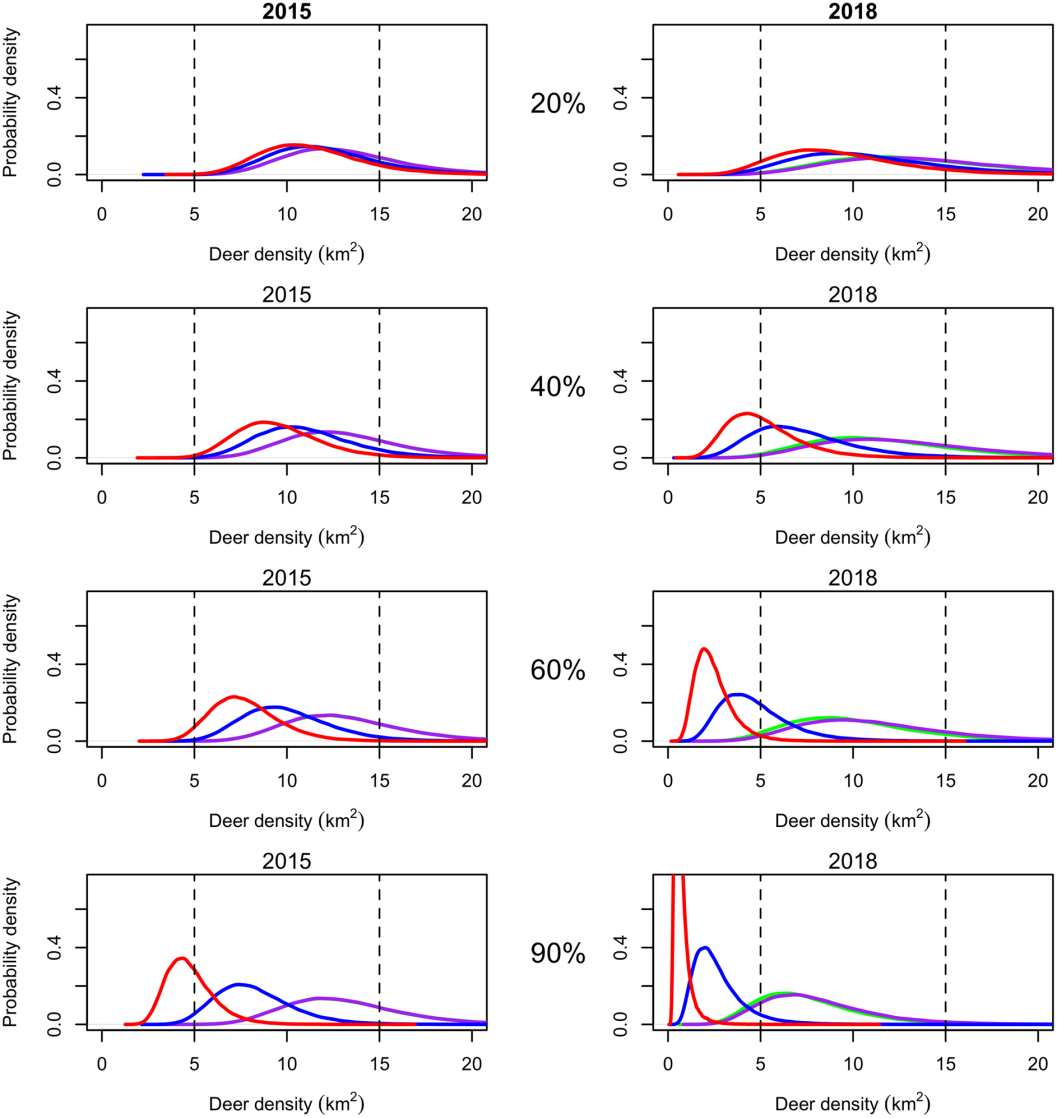
Effects of fertility control treatments on white-tailed deer population densities forecasted for 2015 and 2018 after culling to a management objective or 10 deer/km^2^ in 2014 followed by fertility control treatment. The rows corresponding to the percent of adult females treated from top to bottom: 20%, 40%, 60%, and 90%. The black dotted vertical line represents population density after culling in 2014 to give a baseline for population growth. The dashed vertical lines represent the bounds of the hypothetical management objective. The purple distribution represents the population effect of contraceptives with one year effectiveness which has the potential to maintain the population. The green distribution represents the population effect of contraceptives with three year effectiveness which has a similar effect to the 1 year contraceptives. The blue distribution represents sterilization. Sterilization reduced the population at a faster rate than contraceptives. The red distribution represents culling. Continuing to cull after an initial cull of 90% of the population further decreases the population beyond the management objective.

**Fig 11:**
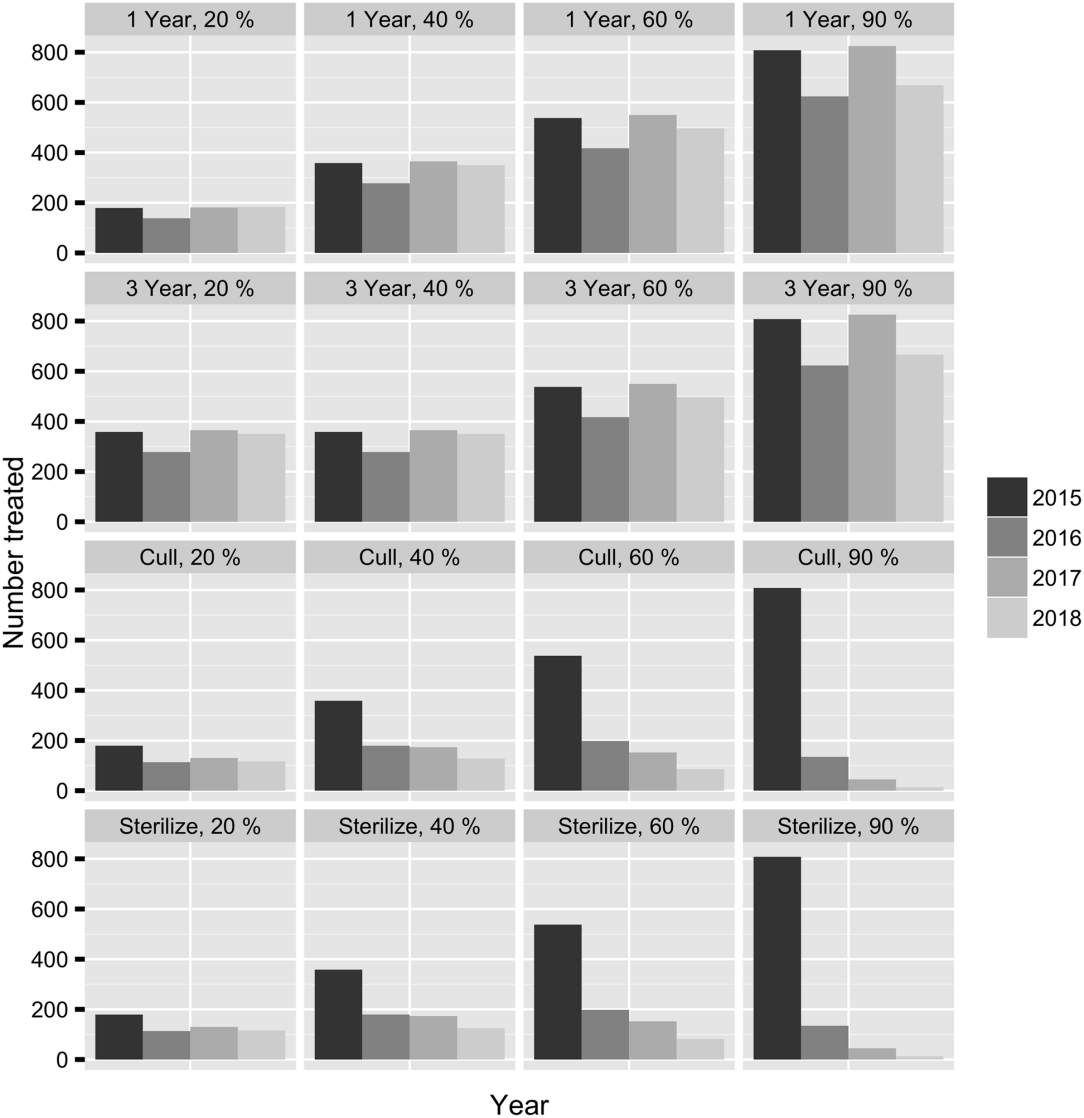
The median number of adult females that would need to be treated across all parks after a 90% population reduction for each management scenario over four years.

*Maintaining a population with fertility control*.—Culling caused the largest reduction in forecasted deer population density, but culling more than 40% each year led to a high probability that the population would decrease to < 5 deer per km^2^ (Fig 8). Contraceptives had a very small probability of reducing the population relative to no action even at high treatment levels and required impractical treatment numbers (Fig 9). It may be desirable to reduce a population initially by culling and then maintain it at a reduced level with fertility control. We chose model experiments that represented the best case scenario for management and evaluated culling 90% the first year followed by treating 20%, 40%, 60%, and 90% of the population with sterilization, one and three year duration contraceptives (Fig 10 and Table B in S1 File). Sterilization and contraceptives of one and three year efficacy had high probabilities of maintaining the population within the management objective especially when administered at lower levels. Treatment numbers were much lower when the objective was to maintain the population with fertility control versus decrease it (Fig 11). However, treatment numbers for 1 and 3 year contraceptives at the 20% level after culling 90% (≈ 1,400 over 5 years) were more than twice the treatment numbers for sterilizing 20% after culling 90% (≈ 540 over 5 years). Sterilizing 20% after culling 90% had a high probability of maintaining the population the first few years but then had an increasing probability of driving the population below the management objective. Sterilization and culling have similar treatment numbers, but culling at any level after a drastic reduction has a higher probability of decreasing the population too much. In every case, implementing a fertility control regime after culling to a desirable level greatly reduced the number of adult female deer that needed to be treated and increased the probability of maintaining the population within the management objective (Figs 10 and 11).

## Discussion

### Population Vital Rates

Posterior distributions of adult female survival (0.67, SD = 0.072) closely resembled results we calculated from published literature (0.74, SD = 0.14) [42–49]. Our estimate for juvenile survival (0.75, SD = 0.1) was also very similar to the average from previous research (0.67, SD = 0.20) [42, 43, 46, 48–52]. Mean adult male survival probability estimated in our model (0.45, SD = 0.096) was also similar to the adult male survival probabilities found in the literature (0.39, SD = 0.19) [43, 46, 48, 53].

Our model estimated the maximum birth rate of white-tailed deer to be *r*_*f*_ = 0.86 with an SD = 0.12. The number of juvenile per adult female at low densities has typically been reported to be near 0.98 [25] or 1.30 [46]. Our estimate for carrying capacity was also lower than the current regional median (*K*_*f*_ = 27, SD = 2.5). It is important to represent density dependence in this population model of white-tailed deer because culling a population may elevate recruitment [54]. We observed density dependence but our parameter estimates are much lower than expected. There are three reasons why this may have happened. First, the birth rates reported by [25] and [46] do not include neonatal survival. We estimated neonatal survival as part of maximum birth rate because of the timing of the birth pulse and the census. Second, our model estimated a lower than expected maximum birth rate to account for low numbers of births observed in the data. Finally, our model did not differentiate between female adults and yearlings, and yearlings are known to produce fewer fawns [51].

Our model also did not have an explicit term for immigration/emigration because we do not have data to support an estimate for that parameter. Excluding this process from our model can be justified because white-tailed deer exhibit high fidelity to home ranges and reduction in deer density does not lead to increased immigration [55]. However, the parks we studied were small enough that treating their populations as closed may be unrealistic. We caution that failing to include density-dependent immigration may lead to overly optimistic conclusions on the benefits of fertility control. This means that modeling the population as open to immigrants would strengthen the conclusions of the current model indicating that fertility control is an infeasible alternative for reducing population densities.

### Population Growth Rate

Mathematical models have shown theoretically that culling a population to a target density followed by fertility control can maintain the population at that target while treating fewer animals than would need to be culled to meet the same objective [7–9]. Our results suggest that all types of fertility control, including culling, will accelerate the decline in the population. However, the number of individuals that need to be treated to reach a stated objective (i.e., relative effort) differs greatly between contraceptives (1 or 3 year efficacy) and culling/sterilization. We confirm the original, theoretical prediction that culling may be used to reduce a population to a target density followed by maintaining the population with contraceptives with a lower relative effort than using contraceptives or culling alone. However, we also caution that density dependent immigration could make it difficult or impossible to maintain populations at low densities using fertility control after reducing densities with culling.

The white-tailed deer population in the Washington, D.C. area may decrease or remain stable with no action (*λ* = 0.992, BCI = {0.824, 1.16}). All actions accelerated the current rate of decline. Contraceptives with an average of three year effectiveness had a slightly larger effect on population growth rate than contraceptives with an average of one year effectiveness, causing the population to decrease by 50% in 6 years if survival probabilities remain constant. Using contraceptives alone has a higher variance and a slower effectiveness than sterilization unless a large fraction of the population can be treated with contraceptives (> 90%). Sterilization also decreased the population growth rate (0.7). Culling had the most dramatic effect on population growth rate, causing the population to decrease by more than 50% in 1 year if 90% of the adult female population were culled.

### Management Implications

Managers often seek to reduce populations of overabundant ungulates. It has been suggested that fertility control could offer an attractive alternative to lethal controls on abundance. Our work casts doubt on the feasibility of fertility control as a way to control overabundance.

We show that it is possible to reduce densities of populations of white-tailed deer with contraceptives, but only if 90% of females are treated for several sequential years. The number of adult female deer that would need to be given contraceptives to reduce the population rapidly would exceed 12,900 over 5 years throughout all parks. The cost per doe for non-surgical fertility control is estimated to be $750. Implementing this management regime would cost over $9,675,000 with a average probability of achieving the objective (1 year contraceptives Probability in = 0.5, 3 year contraceptives Probability in = 0.65). Sterilization has an average to high probability of success if between 40% (Probability in = 0.57) and 60% (Probability in = 0.81) of adult females are treated each year, but the costs would be more than double the cost of contraceptives.

Culling is less expensive than administering contraceptives. The cost per doe for culling is estimated to be $370. Only 3,510 adult females (40%) need to be culled over 5 years ($1,298,700) to have a probability of 0.91 of being within the management objective. Assuming our hypothetical management objective, culling 40% of females each year is 9.1% more likely to achieve the management objective than administering contraceptives to 90% of females each year.

Each of the management strategies has its own set of undesirable effects. Culling alone can cause a population to fall below a desirable management objective and in extreme cases cause a closed population to become locally extinct. Regulated harvests can be unpopular with the public or logistically complicated due to proximity to urban areas. However, reducing the survival of females by culling decreases the relative effort required to achieve the same goal by contraceptives or sterilization. Using contraceptives after culling to a desired management objective may be an effective way to maintain a population below carrying capacity because fewer animals need to be culled and fewer animals need to be treated with contraceptives (Table B in S1 File). If 90% of adult females were culled the first year followed by 1 year contraceptives administered to 20% for 5 years, the probability that the population would be within the management objective is 0.62. A hypothetical treatment plan may be as follows: 3,200 adult females would need to be culled the first year ($1,184,000) and 680 would need to be administered contraceptives over the next four years ($510,000). This type of management plan reduces the relative effort 82% compared to using only fertility control and maintains the probability that the population will remain below carrying capacity but will not fall to unacceptably low levels.

Effective methods for regulating overabundant ungulates are needed in many ecosystems [56, 57]. We provided evidence that contraceptives alone will reduce the population growth of white-tailed deer relative to taking no action but will not reduce abundance rapidly unless > 90% of reproductive females can be treated. Sterilization has the potential to decrease the population and maintain it below carrying capacity if 40% of adult females can be treated each year. However, if the objective is to reduce the population rapidly, the relative effort of implementing these fertility control regimes outweighs the probability that the management objective will be achieved unless there is an initial cull. We have confirmed with data and a rigorous assessment of uncertainty the theoretical prediction that culling to a predetermined level followed by administering lower amounts of fertility control, rather then implementing a contraceptive program alone, may be an efficient way to achieve management objectives for maintaing populations below carrying capacity with low relative effort.

## Supporting Information

S1 File Supporting appendices, figures, and tables. (ZIP)

S2 File Code and data files. (ZIP)
