# Supplementary material for: Forecasting the Effects of Fertility Control on Overabundant Ungulates: White-Tailed Deer in the National Capital Region
Source: PLoS One. 2015 Dec 9;10(12):e0143122. doi: 10.1371/journal.pone.0143122 (PMC4674220; doi:10.1371/journal.pone.0143122)
Supplement: S1 File — (ZIP) [file pone.0143122.s001.zip › S1_File.pdf]

Forecasting the Effects of Fertility Control on Overabundant Ungulates: White-tailed Deer in the National Capital Region

Ann M. Raiho[1,*], Mevin B. Hooten[2], Scott Bates[3], N. Thompson Hobbs[1]

1 Natural Resource Ecology Laboratory, Department of Ecosystem Science and Sustainability, and Graduate Degree Program in Ecology, Colorado State University, Fort Collins CO, 80523, USA
2 U.S. Geological Survey, Colorado Cooperative Fish and Wildlife Research Unit; Department of Fish, Wildlife, and Conservation Biology and Department of Statistics, Colorado State University, Fort Collins, CO 80523, USA
3 Urban Ecology Center, National Capital Region, National Park Service, Washington, D.C., USA.

*** Contact Information: araiho@nd.edu, 100 Galvin Life Sciences Center, Notre Dame, IN 46556**

Supporting Information

Appendix A. The Full Posterior Distribution.

$$
\begin{aligned}
\left[\boldsymbol{\theta}, \boldsymbol{n}_{it}, \sigma^2 \,\middle|\, \boldsymbol{y}_{\boldsymbol{\alpha}_{it}}, y_{d_{it}}, \hat{\sigma}_{it}, y_{N_{it}}\right] \propto\; & \prod_{i=1}^{8}\prod_{t=1}^{13}\left(\left[\boldsymbol{y}_{\boldsymbol{\alpha}it} \mid y_{N_{it}}, \boldsymbol{\pi}_{it}\right]\right) \\
\times\; & \prod_{i=1}^{8}\prod_{t=1}^{13}\left(\left[y_{d_{it}} \,\middle|\, \frac{\sum_{j=1}^{3} n_{jit}}{area_i}, \hat{\sigma}^2_{it}\right]\left[\log\left(\boldsymbol{n}_{it}\right) \mid \log\left(\mathbf{A}_{it-1}\mathbf{n}_{it-1}\right), \sigma^2_p \mathrm{I}\right]\right) \\
\times\; & \prod_{i=1}^{8}\left(\left[d_{i1} \mid y_{d_{i1}}, \hat{\sigma}^2_{i1}\right]\left[\boldsymbol{\gamma}_{i1} \mid \boldsymbol{y}_{\boldsymbol{\alpha}_{i1}} + 1\right]\right) \\
\times\; & \prod_{i=1}^{8}\prod_{j=1}^{3}\left[s_{ij}|a_j, b_j\right]\left[r_f|2\cdot 3.09w^{-0.33}, .1304^2\right]\left[\mathrm{K_f}|0,100\right]\left[m|312,312\right]\left[\sigma^2|0,2\right] \\
\times\; & \prod_{j=1}^{3}\left[a_j|\frac{\mu^2_{a_j}}{\sigma^2_{a_j}}, \frac{\mu_{a_j}}{\sigma^2_{a_j}}\right]\left[b_j|\frac{\mu^2_{b_j}}{\sigma^2_{b_j}}, \frac{\mu_{b_j}}{\sigma^2_{b_j}}\right]
\end{aligned}
$$

Appendix B. Mathematical Descriptions of Model Experiments.

In the culling experiment c represents the proportion of adult females that were culled. The projection matrix for the culling experiment was

$$\begin{bmatrix} 0 & s_2 f(1-c) & 0 \\ s_1 m & s_2(1-c) & 0 \\ s_1(1-m) & 0 & s_3 \end{bmatrix} \cdot \begin{bmatrix} n_1 \\ n_2 \\ n_3 \end{bmatrix}_t = \begin{bmatrix} n_1 \\ n_2 \\ n_3 \end{bmatrix}_{t+1} \tag{1}$$

We created an additional state including infertile females (n_4) to represent treatment with fertility control agents. The parameter c represents the proportion of fertile adult females annually treated. The model for sterilization (permanent infertility) was

$$\begin{bmatrix} 0 & s_2 f(1-c) & 0 & 0 \\ s_1 m & s_2(1-c) & 0 & 0 \\ s_1(1-m) & 0 & s_3 & 0 \\ 0 & s_2 c & 0 & s_2 \end{bmatrix} \cdot \begin{bmatrix} n_1 \\ n_2 \\ n_3 \\ n_4 \end{bmatrix}_t = \begin{bmatrix} n_1 \\ n_2 \\ n_3 \\ n_4 \end{bmatrix}_{t+1} \tag{2}$$

Treatment with single year contraceptives was modeled using

$$\begin{bmatrix} 0 & s_2 f & 0 & 0 \\ s_1 m & s_2(1-c) & 0 & s_2(1-c) \\ s_1(1-m) & 0 & s_3 & 0 \\ 0 & s_2 c & 0 & s_2 c \end{bmatrix} \cdot \begin{bmatrix} n_1 \\ n_2 \\ n_3 \\ n_4 \end{bmatrix}_t = \begin{bmatrix} n_1 \\ n_2 \\ n_3 \\ n_4 \end{bmatrix}_{t+1} \tag{3}$$

The fecundity term for adult females (n_3) was not influenced by this treatment because it does not affect the viability of a current pregnancy.

For fertility control with longer efficacy than one year, we represented a hypothetical contraceptive agent that on average renders animals infertile for three years. Let α be the average duration of a fertility control agent. It follows that the probability that a treated female becomes fertile during each year following treatment is ψ, where $\psi = 1 - e^{-\alpha \triangle t}, \alpha^{-1} = 3$. Thus, the model for the three year treatment was

$$\begin{bmatrix} 0 & s_2 f & 0 & 0 \\ s_1 m & s_2(1-c) & 0 & s_2(1-c)(1-\psi) \\ s_1(1-m) & 0 & s_3 & 0 \\ 0 & s_2 c & 0 & s_2 c + s_2(1-c)\psi \end{bmatrix} \cdot \begin{bmatrix} n_1 \\ n_2 \\ n_3 \\ n_4 \end{bmatrix}_t = \begin{bmatrix} n_1 \\ n_2 \\ n_3 \\ n_4 \end{bmatrix}_{t+1} \tag{4}$$

Appendix C. Supporting Tables and Figures.

Figure A. Posterior Distributions of Matrix Sensitivities. The posterior distributions of the matrix sensitivities.

Figure B. Posterior Predictive Checks. Test statistics for posterior predictive checks were calculated from observed data ($T^{obs} = \sum_{i=1}^{I} \sum_{t=1}^{T} (y_{d_{it}} - \mu_{it})^2$) and from simulated data ($T^{rep} = \sum_{i=1}^{I} \sum_{t=1}^{T} \left(y_{d_{it}}^{rep} - \mu_{it}\right)^2$) where y_d^{rep} is a dataset drawn from the posterior predictive distribution at each iteration of the MCMC algorithm and μ_{it} is the model prediction of the median of the distribution of the density of white-tailed deer in each park, each year. The Bayesian P value is
$P_B = Pr\left[T^{rep}(y^{rep}, \theta) \geq T^{obs}(y, \theta) \mid y\right]$. This image was thinned by 10 to enhance clarity of the plot.

Table A. Estimated Number Treated for Model Experiment without Initial Cull. We report the effects of four management actions (culling, sterilization, 1 year contraceptives, and 3 year contraceptives) at four different levels (20%, 40%, 60%, and 90% treated). For each treatment, we calculated the probability that the population will be below the objective (P <), within the objective (P in), and above the objective (P >). We also show the median number of adult females that would need to be treated throughout all parks.

Treatment	Percent Treated	Year	P <	P in	P >	Number Treated
No Action		2014	0.00	0.00	1.00	0
		2015	0.00	0.00	1.00	0
		2016	0.00	0.00	1.00	0
		2017	0.00	0.01	0.99	0
		2018	0.00	0.02	0.98	0
3 Year	20	2014	0.00	0.00	1.00	717
		2015	0.00	0.00	1.00	601
		2016	0.00	0.00	1.00	614
		2017	0.00	0.01	0.99	568
		2018	0.00	0.04	0.96	546
	40	2014	0.00	0.00	1.00	1436
		2015	0.00	0.00	1.00	1213
		2016	0.00	0.01	0.99	1237
		2017	0.00	0.03	0.97	1106
		2018	0.00	0.09	0.91	1042
	60	2014	0.00	0.00	1.00	2151
		2015	0.00	0.00	1.00	1823
		2016	0.00	0.02	0.98	1858
		2017	0.00	0.10	0.90	1593
		2018	0.00	0.24	0.76	1466
	90	2014	0.00	0.00	1.00	3231
		2015	0.00	0.00	1.00	2726
		2016	0.00	0.08	0.92	2779
		2017	0.00	0.37	0.63	2223
		2018	0.00	0.65	0.35	1990
1 Year	20	2014	0.00	0.00	1.00	717
		2015	0.00	0.00	1.00	602
		2016	0.00	0.00	1.00	612
		2017	0.00	0.01	0.99	564
		2018	0.00	0.02	0.98	551
	40	2014	0.00	0.00	1.00	1436
		2015	0.00	0.00	1.00	1211
		2016	0.00	0.00	1.00	1233
		2017	0.00	0.02	0.98	1104
		2018	0.00	0.04	0.96	1066
	60	2014	0.00	0.00	1.00	2151
		2015	0.00	0.00	1.00	1821
		2016	0.00	0.01	0.99	1853
		2017	0.00	0.05	0.95	1597
		2018	0.00	0.10	0.90	1518
	90	2014	0.00	0.00	1.00	3231
		2015	0.00	0.00	1.00	2726
		2016	0.00	0.06	0.94	2782
		2017	0.00	0.28	0.72	2229

		2018	0.00	0.50	0.49	2031
Sterilize	20	2014	0.00	0.00	1.00	717
		2015	0.00	0.00	1.00	506
		2016	0.00	0.02	0.98	431
		2017	0.00	0.06	0.94	347
		2018	0.00	0.14	0.86	291
	40	2014	0.00	0.00	1.00	1436
		2015	0.00	0.01	0.99	827
		2016	0.00	0.10	0.90	566
		2017	0.00	0.33	0.67	371
		2018	0.00	0.57	0.43	260
	60	2014	0.00	0.00	1.00	2151
		2015	0.00	0.06	0.94	959
		2016	0.00	0.41	0.59	484
		2017	0.01	0.74	0.26	243
		2018	0.08	0.81	0.10	133
	90	2014	0.00	0.00	1.00	3231
		2015	0.00	0.41	0.59	808
		2016	0.02	0.80	0.18	135
		2017	0.22	0.72	0.06	35
		2018	0.50	0.47	0.03	11
Cull	20	2014	0.00	0.00	1.00	717
		2015	0.00	0.01	0.99	505
		2016	0.00	0.07	0.93	430
		2017	0.00	0.18	0.82	354
		2018	0.00	0.32	0.68	305
	40	2014	0.00	0.00	1.00	1436
		2015	0.00	0.18	0.82	826
		2016	0.00	0.59	0.41	566
		2017	0.00	0.85	0.15	393
		2018	0.04	0.91	0.05	293
	60	2014	0.00	0.06	0.94	2151
		2015	0.00	0.83	0.17	959
		2016	0.04	0.96	0.01	484
		2017	0.41	0.59	0.00	272
		2018	0.82	0.18	0.00	164
	90	2014	0.00	0.86	0.14	3231
		2015	0.64	0.36	0.00	809
		2016	0.98	0.02	0.00	135
		2017	1.00	0.00	0.00	45
		2018	1.00	0.00	0.00	13

Table B. Estimated Number Treated for Model Experiment without Initial Cull. We implemented four management actions (culling, sterilization, 1 year contraceptives, and 3 year contraceptives) at four different levels (20%, 40%, 60%, and 90% treated) after culling 90% of the adult female population. For each treatment, we calculated the probability that the population will be below the objective (P <), within the objective (P in), and above the objective (P >). We also show the median number of adult females that would need to be treated throughout all parks.

Treatment	Percent Treated	Year	P <	P in	P >	Number Treated
3 Year	20	2015	0.00	0.75	0.25	358
		2016	0.00	0.75	0.24	278
		2017	0.00	0.70	0.30	365
		2018	0.01	0.66	0.33	350
	40	2015	0.00	0.75	0.25	358
		2016	0.00	0.81	0.19	278
		2017	0.01	0.79	0.21	365
		2018	0.01	0.78	0.21	350
	60	2015	0.00	0.75	0.25	538
		2016	0.00	0.86	0.14	417
		2017	0.01	0.86	0.13	550
		2018	0.04	0.86	0.10	496
	90	2015	0.00	0.75	0.25	808
		2016	0.01	0.92	0.07	623
		2017	0.04	0.90	0.06	826
		2018	0.17	0.81	0.02	667
1 Year	20	2015	0.00	0.75	0.25	179
		2016	0.00	0.76	0.24	138
		2017	0.00	0.68	0.32	181
		2018	0.00	0.62	0.37	183
	40	2015	0.00	0.75	0.25	358
		2016	0.00	0.81	0.19	278
		2017	0.00	0.75	0.25	365
		2018	0.01	0.72	0.27	350
	60	2015	0.00	0.75	0.25	538
		2016	0.00	0.86	0.14	417
		2017	0.01	0.82	0.17	550
		2018	0.02	0.82	0.16	497
	90	2015	0.00	0.75	0.25	808
		2016	0.01	0.93	0.07	624
		2017	0.03	0.90	0.07	825
		2018	0.13	0.84	0.03	669
Sterilize	20	2015	0.00	0.85	0.15	179
		2016	0.00	0.86	0.14	113
		2017	0.01	0.84	0.14	130
		2018	0.03	0.82	0.15	116
	40	2015	0.00	0.92	0.08	358
		2016	0.02	0.94	0.04	179
		2017	0.08	0.89	0.03	173
		2018	0.20	0.78	0.02	125
	60	2015	0.00	0.96	0.04	538
		2016	0.08	0.91	0.01	198
		2017	0.35	0.65	0.00	152
		2018	0.63	0.37	0.00	82

	90	2015	0.03	0.96	0.01	808
		2016	0.46	0.54	0.00	135
		2017	0.82	0.18	0.00	45
		2018	0.94	0.06	0.00	13
Cull	20	2015	0.00	0.90	0.10	179
		2016	0.01	0.92	0.08	114
		2017	0.03	0.89	0.07	130
		2018	0.07	0.86	0.07	117
	40	2015	0.00	0.97	0.02	358
		2016	0.09	0.90	0.01	179
		2017	0.31	0.69	0.00	174
		2018	0.54	0.46	0.00	128
	60	2015	0.04	0.96	0.00	538
		2016	0.48	0.52	0.00	198
		2017	0.88	0.12	0.00	152
		2018	0.98	0.02	0.00	85
	90	2015	0.63	0.37	0.00	808
		2016	0.98	0.02	0.00	135
		2017	1.00	0.00	0.00	45
		2018	1.00	0.00	0.00	13

Data Accessibility.

White-tailed deer data are property of the United States government and is publicly available in the NPS Data Store located at https://irma.nps.gov/. The data used for this particular analysis and R scripts used to implement the model are located at https://github.com/araiho/Deer and can also be found in the supplementary materials of this paper.
