# Supplementary figures and images for: Forecasting the Effects of Fertility Control on Overabundant Ungulates: White-Tailed Deer in the National Capital Region
Source: PLoS One. 2015 Dec 9;10(12):e0143122. doi: 10.1371/journal.pone.0143122 (PMC4674220; doi:10.1371/journal.pone.0143122)

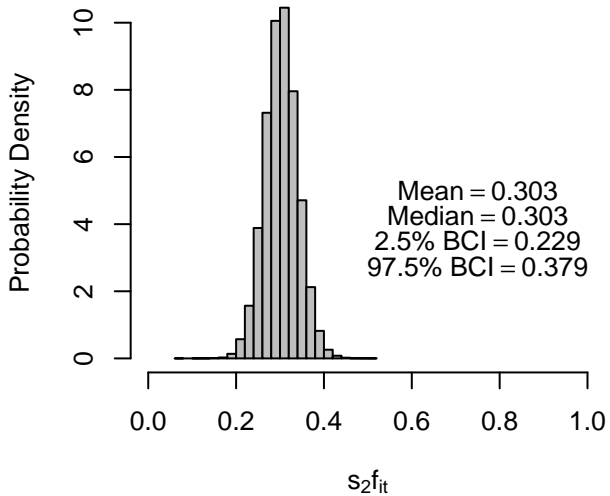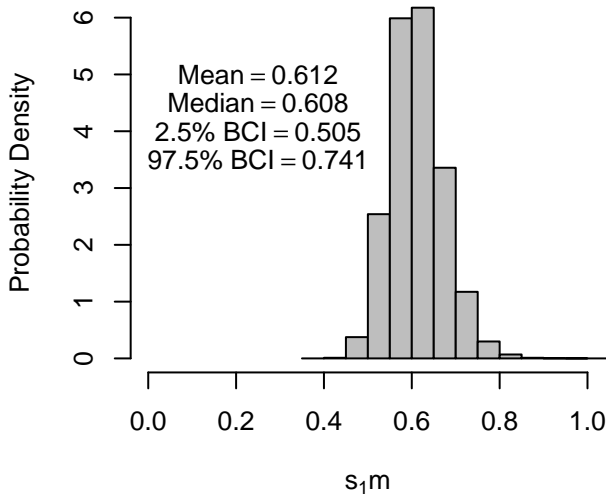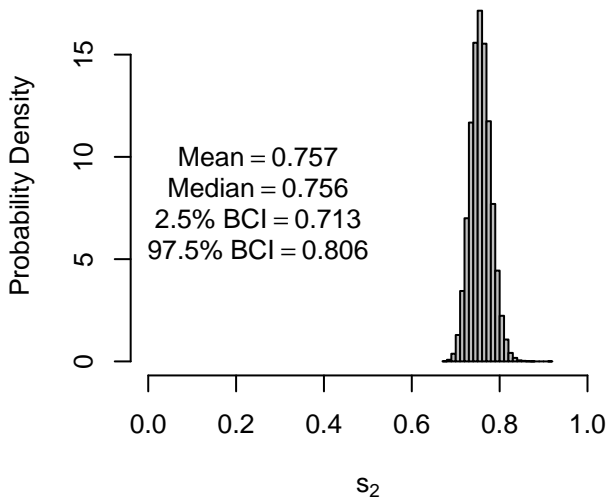

Supplement: S1 File — (ZIP) [file pone.0143122.s001.zip › S1_FigureA.pdf]

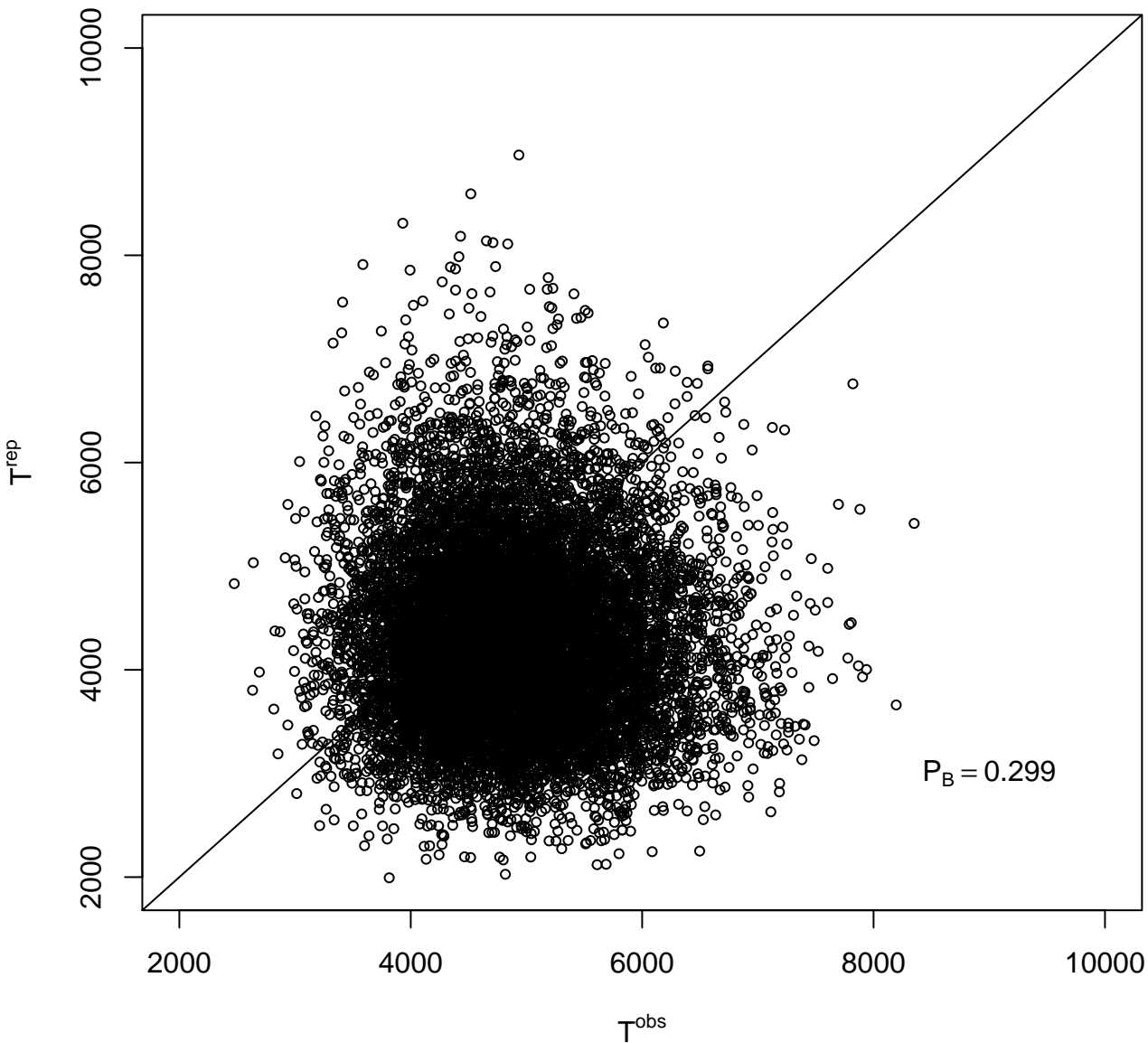

Supplement: S1 File — (ZIP) [file pone.0143122.s001.zip › S1_FigureB.pdf]
