# Supplementary material for: Forecasting the Effects of Fertility Control on Overabundant Ungulates: White-Tailed Deer in the National Capital Region
Source: PLoS One. 2015 Dec 9;10(12):e0143122. doi: 10.1371/journal.pone.0143122 (PMC4674220; doi:10.1371/journal.pone.0143122)
Supplement: S2 File — (ZIP) [file pone.0143122.s002.zip › Data_and_Code/Distance Methods Summary Form and Detailed Form.docx]

**Methods**

Field methods for collecting Distance data and analyzing the data followed NCR Distance Protocols described in the monitoring plan for the region (NPS NCRN 2005). All 2014 analyses were done by Wildlife Resources Program. Spotlight data was entered into Distance software (Thomas et al. 2006).

Most parks were surveyed during fall 2014 for at least three nights (Table 1). MANA and MONO were surveyed for two nights; GREE, FTWA, PISC and PRWI were sampled for 4 nights. Each night was treated as a replicate, and the data were pooled for analysis.

For the initial analysis, the detections were divided into 10-12 evenly-divided distance intervals. Intervals were expanded, narrowed, or dropped from the analysis to produce a smooth shoulder as the distance from the observer to the deer increased. A smooth shoulder prevents heaping of data into one distance interval, and corrects for outliers cause by evasive movements (Buckland et al. 2001). Once a satisfactory shoulder was produced, four models were fit to the data (uniform, half-normal, hazard rate, and negative exponential). The three criteria used to choose the best fitted model were: (1) percent coefficient of variation (CV) less than 20; (2) the detection probability variation was less than 30%; and (3) lowest Akaike’s Information Criterion (AIC) score. Program Distance calculates all three measures.

**Table 1**. Survey history of parks using Distance.

| **Park** | **Park Code** | **Number of Fall Surveys – 2014** | **Year of First Fall Survey** |
| --- | --- | --- | --- |
| Antietam | ANTI | 3 | 2001 |
| Monocacy | MONO | 1 | 2001 |
| Piscataway | PISC | 4 | 2001 |
| Greenbelt | GREE-NACE | 3 | 2001 |
| Manassas | MANA | 2 | 2000 |
| Catoctin | CATO | 5 | 2000 |
| Great Falls | GWMP | 3 | 2001 |
| Great Falls | CHOH | 3 | 2000 |
| Prince William | PRWI | 4 | 2001 |
| Rock Creek  Fort Washington | ROCR  FTWA-NACE | 4  3 | 2000  2009 |

DISTANCE ANALYSIS PROCEDURE

1. Key in and validate the data.
2. Click on the ‘inputs’ tab and then the data filter ‘properties’ tab to specify the distance cutpoints. You want about 10-15 cutpoints. Click ‘Data Selection”and check the box to transform distance data into intervals. Click ‘Automatic equal intervals’ and specify the number of intervals. Make sure that the longest distance equals or exceeds the longest recorded survey distance. Then click the model definition ‘properties’ tab to specify the key functions and model expansions.

Start with the ‘Estimate’ tab. Click ‘no stratification’ to analyze the entire survey. This should ensure that the ‘Global’ level of resolution is selected for the variables to be estimated.

Then click ‘Detection function’. Click the’+’ tab to specify the number of models to run. The key function and corresponding expansion is as follows: half normal/hermite; uniform/simple; uniform/polynomial; hazard rate/cosine. You can run the negative exponential key function as well but I have never seen a recommended expansion so if I’m not satisfied with the first run of models above, I will run the negative exponential with all of the expansions.

Click ‘cluster size’ tab and click on the ‘size bias’ cluster size estimation method.

Click the ‘regress ln(cluster size) against estimated gx.

Click the ‘variance’ tab to make sure the variance is calculated empirically.

Click the ‘run’ button to run the model.

Click the ‘results’ tab and then the ‘Estimation Options Listing’ drop down arrow; select the Density Estimates/Global option. If the D CV is less than 20% and the detection probability percentage of the variation is less than 30%, the model should be accepted as is. If either of these 2 values are above these thresholds, the model distance cutpoints have to be changed.

Check for the lack of a shoulder around the transect line and spikes that are far from the transect line. Use different cutpoints for the distance intervals to create a shoulder around the transect line and/or reduce the spikes to smooth out the shape of the histogram. If there are a lot of detections far from the transect line you can start by truncating 5% of the longest distances.

1. I prefer to use the CV and detection probability variance as my indicators for the best model. The model with the lowest AIC tends to have the highest density and I believe it overestimates the population.

The chi-sq GOF test can be used to evaluate the shoulder of the model but is considered a weak test.

If one model does not stand out you can average the models but until 2013 I have never done this.
