# Supplementary material for: Forecasting the Effects of Fertility Control on Overabundant Ungulates: White-Tailed Deer in the National Capital Region
Source: PLoS One. 2015 Dec 9;10(12):e0143122. doi: 10.1371/journal.pone.0143122 (PMC4674220; doi:10.1371/journal.pone.0143122)
Supplement: S2 File — (ZIP) [file pone.0143122.s002.zip › Data_and_Code/USGS_Disclaimers.txt]

Software Disclaimer:Although this software program has been used by the U.S. Geological Survey (USGS), no warranty, expressed or implied, is made by the USGS or the U.S. Government as to the accuracy and functioning of the program and related program material nor shall the fact of distribution constitute any such warranty, and no responsibility is assumed by the USGS in connection therewith.Data Disclaimer:Although these data have been processed successfully on a computer system at the U.S. Geological Survey (USGS), no warranty expressed or implied is made regarding the display or utility of the data on any other system or for general or scientific purposes, nor shall the act of distribution constitute any such warranty. The USGS or the U.S. Government shall not be held liable for improper or incorrect use of the data described and/or contained herein.
